# THE READYR PROGRAM'S INTERIM FEASIBILITY, ACCEPTABILITY, AND EFFECTS ON VALUES-BASED CARE AND RELATIONSHIP QUALITY

**DOI:** 10.1093/geroni/igac059.1547

**Published:** 2022-12-20

**Authors:** Shirin Hiatt, Joel Steele, Chao-Yi Wu, Lyndsey Miller

**Affiliations:** Oregon Health & Science University, Portland, Oregon, United States; Portland State University, Portland, Oregon, United States; Oregon Health & Science University, Portland, Oregon, United States; Oregon Health & Science University, Portland, Oregon, United States

## Abstract

This study evaluated preliminary feasibility, acceptability and efficacy of the READyR program, a remote intervention for early stage dementia dyads designed to assess and address values-based care and relationship quality. Measures of program acceptability, preparation for future care, and relationship quality were examined pre- and post-intervention. Feasibility was high among the first 10 enrolled dyads who completed phase 1: 39 out of 40 sessions were completed with 100% retention. Acceptability was also high with nearly 95% reporting that participation was easy and that they would recommend it to others. Insufficient time was the only critique reported by 17%. Medium/large effects were found in improvements to positive interactions (d=-0.71 95%CI [-1.34-.00]), relationship strain (d=0.53 95%CI [.-.15-1.18]), and awareness (d=-0.91 95%CI [-1.64- -0.15]) and avoidance (d=-0.53 95%CI [-1.18-.15]) of preparation for future care needs. The READyR program shows preliminary feasibility, acceptability, and efficacy in improving values-based care and relationship quality remotely.

